# De Novo Interstitial Microdeletion at 1q32.1 in a 10-Year-Old Boy with Developmental Delay and Dysmorphism

**DOI:** 10.1155/2016/2501741

**Published:** 2016-02-03

**Authors:** Jennifer Carter, Melinda Zombor, Adrienn Máté, László Sztriha, Jonathan J. Waters

**Affiliations:** ^1^NE Thames Regional Genetics Service, Great Ormond Street Hospital NHS Foundation Trust, Barclay House Levels 5&6, 37 Queen Square, London WC1N 3BG, UK; ^2^Division B, Department of Paediatrics, University of Szeged, Temesvári Körút 35-37, Szeged 6726, Hungary

## Abstract

A 10-year-old boy was referred with developmental delay and dysmorphism. Genomewide aCGH microarray analysis detected a de novo 3.7 Mb deletion at 1q32.1: arr 1q32.1(199,985,888-203,690,832)x1 dn [build HG19]. This first report of a deletion in this region implies a critical role for dosage-sensitive genes within 1q32.1 in neurological development. This is consistent with previously reported duplications of this region in patients with a similar phenotype.

## 1. Introduction

Developmental delay with or without dysmorphism is the most common referral for microarray analysis in the cytogenetic laboratory; and microarray analysis is a critical tool for identifying copy number changes in these patients. Deletions of 1q32.1 have not previously been reported in the literature.

## 2. Case Presentation

**Figure 1:**
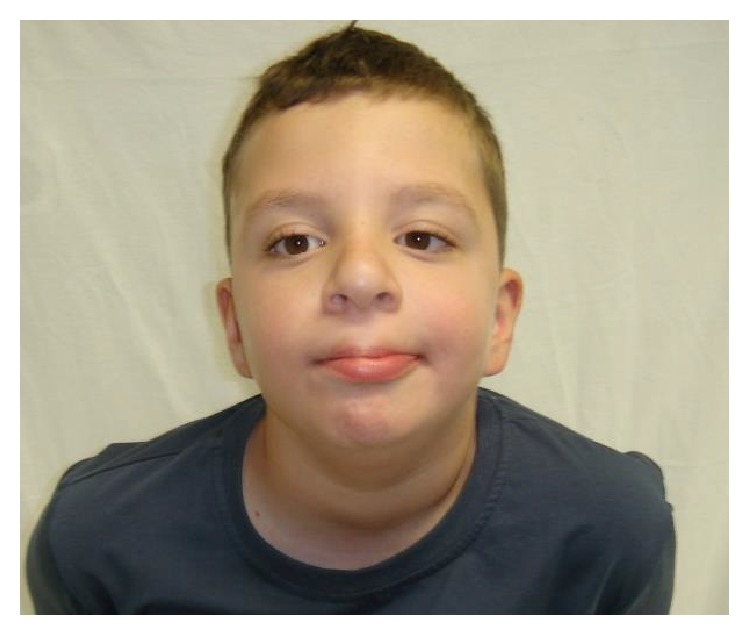
The patient at 10 years of age.

The patient was presented for genetic testing at 10 years of age with dysmorphic features and intellectual disability (Figure 1). He was born at term from a first uneventful pregnancy without perinatal complications. Birth weight was 3450 g (50th centile), length 51 cm (50th centile), and head circumference 35 cm (50th centile). At 12 months of age he had global developmental delay and was able to sit. He was able to walk at 18 months but had difficulties with social skills.

On examination at the age of 4 years, his head circumference was 51 cm (50th centile) and the other growth parameters were also normal. Dysmorphic features included a long face, narrow jaw, downslanted palpebral fissures, highly arched eyebrows, low-set ears, thick lower lip, bilateral fifth finger clinodactyly, and proximal placement of the thumb. Neurological assessment revealed generalized hypotonia and reduced deep tendon reflexes.

Neuropsychological evaluation was performed at 7 years of age. A full scale IQ of about 50 was established by the Woodcock-Johnson Tests of Cognitive Abilities, and the Bender Visual Gestalt Test revealed difficulties in visual-motor coordination. Speech evaluation indicated significant difficulties with receptive and expressive language; however a slow improvement in language acquisition has been observed. At the age of 10 years he requires special education and support in everyday life.

Routine EEG and brain MRI were normal. Fragile X syndrome testing, karyotype, and screening for inborn error of metabolism by blood MS/MS and urine GC/MS, serum CK, and lactate/pyruvate were all normal.

Both parents are healthy and have no other children. There is no history of intellectual disability in the family.

Genomewide array CGH analysis using the Nimblegen 135 K WG CGH v3.1 platform (with a minimal resolution of 0.2 Mb) identified a 3.7 Mb copy number loss on the long arm of chromosome 1: arr 1q32.1(199,985,888-203,690,832)x1 [build HG19]. DNA was extracted from blood samples using the Nucleon (Amersham) and Genomic DNA Purification kits (Gentra) according to the manufacturer's instructions. The microarray was washed and then scanned on an Axon GenePix 4400A Scanner using GenePix Pro 7 software (Molecular Devices, Sunnyvale, CA, USA). Raw data was normalized and LOESS correction applied and the data ratios were calculated using DEVA v1.01 Software (Roche NimbleGen). The normalized data was processed using Infoquant Fusion v6.0 software (Infoquant, London, UK) with analysis call settings of 3 consecutive probes ±0.4 Cy3/Cy5 ratio.

The microarray was carried out using sex matched control DNA and confirmed by qPCR analysis using primers from the ELF3 gene locus and FISH using probe RP11-134G8 (BlueGnome). Parental testing using the same qPCR and FISH probes as the proband showed the deletion to be de novo in origin.

## 3. Discussion

To our knowledge, no deletions of a similar size or location have been reported previously in the literature, and there were no similar-sized deletions found in our internal laboratory database or ECARUCA (European Cytogeneticists Association Register of Unbalanced Chromosome Aberrations). DECIPHER (Database of Chromosomal Imbalance and Phenotype in Humans Using Ensembl Resources, https://decipher.sanger.ac.uk/ (accessed 13/01/2016)) revealed one patient (ref: 259811) with a de novo, smaller (2.59 Mb) deletion but with no phenotypic or predicted pathogenicity information available. A second DECIPHER patient (ref. 288679) was identified with macroglossia, intellectual disability, and a larger (5.95 Mb) deletion of unknown inheritance, partially overlapping with the region of interest in our patient. A third patient was identified from the ISCA (International Standards for Cytogenetic Arrays) database (ref. nssv575720), presenting with developmental delay and a 3 Mb (likely pathogenic) deletion of unknown inheritance. Searching of the DGV (Database of Genomic Variants) indicated that copy number loss is not present in normal individuals.

Duplications within the same region have been associated with global developmental delay, behavioural problems, paraesthesias, staring spells, headaches, motor difficulties, and myoclonic epilepsy [1]. In the Olson study, KDM5B, NAV1, and KIF21B were identified as candidate genes for developmental delay from two patients with microduplications of 1q32.1, and these genes are all common to the deleted region of our patient. They have been reported with an HI index [2] (% haploinsufficiency scores) of 28%, 16%, and 38%, respectively, where the lower the percentage score, the greater the chance that haploinsufficiency will have a phenotypic impact.

In total, 59 genes are located within the deleted region in our patient, of which GPR37L1 and SYT2 (in addition to the three previously mentioned) are expressed in neuronal tissues.

KDM5B is a histone lysine demethylase which is involved in cell maintenance and repair and may have a role in embryonic development [1]. NAV1 is a member of the neuron navigator family which is expressed predominantly in the brain. These navigators are microtubule plus-end tracking proteins which have also been shown to induce the formation of neurite-like extensions in nonneuronal cells and thus have the ability to affect the cytoskeleton [3]. The KIF21B protein is a member of the kinesin family and is a microtubule motor protein also highly expressed in neuronal tissue [1]. GPR37L1 encodes an orphan G protein coupled receptor which is activated by the protein prosaposin. This protein has a protective effect on the neurons and glia in the nervous system [4]; and missense mutations of SYT2 have been associated with myasthenic syndrome, a disorder of the peripheral motor nerve terminal which gives rise to variable limb weakness and reduced deep tendon reflexes [5]. All five of these genes are also deleted in the patient from the ISCA database and from both DECIPHER patients.

It is therefore possible that there is a dosage effect for one or more of these genes which may be causative of the patient's clinical features.

Our literature search did not reveal any genes from this region with a clear association between copy number loss and dysmorphism. Although we cannot exclude unknown gene functions or gene dosage effects, it is possible that this patient's dysmorphism may be coincidental to the copy number loss.

Genes which are expressed in neurological tissues are present in this novel 1q32.1 region of de novo copy number loss. These genes have also been identified in duplications of the same region in patients with a shared phenotype. This implies that for one or more genes in this region, dosage sensitivity as a result of either haploinsufficiency or triplosensitivity impacts on the neurodevelopmental phenotype in ways not yet understood.
